# Willingness to institutionalize a relative with dementia: a web-platform assessment with the Portuguese adapted version of the Desire-to-Institutionalize Scale

**DOI:** 10.3389/fmed.2023.1277565

**Published:** 2024-01-08

**Authors:** Soraia Teles, Milaydis Sosa Napolskij, Oscar Ribeiro, Sara Alves, Alberto Freitas, Ana Ferreira, Constança Paúl

**Affiliations:** ^1^Department of Behavioral Sciences, School of Medicine and Biomedical Sciences, University of Porto (ICBAS-UP), Porto, Portugal; ^2^Center for Health Technology and Services Research of the Associate Laboratory Health Research Network (CINTESIS@RISE), Porto, Portugal; ^3^Faculty of Medicine, University of Porto (FMUP), Porto, Portugal; ^4^Department of Education and Psychology, University of Aveiro, Aveiro, Portugal; ^5^Center of Research, Diagnosis, Training and Care of Dementia (CIDIFAD), SCMRA, Riba D’Ave, Portugal

**Keywords:** informal care, dementia, desire to institutionalize, cultural adaptation, psychometric properties

## Abstract

**Introduction:**

Older persons with dementia (PwD) are more likely to be institutionalized than their counterparts without dementia. The caregiver’s desire to institutionalize has been suggested as the most important predictor of actual institutionalization. This cross-sectional study aimed to culturally adapt the Desire to Institutionalize Scale (DIS) to a country with a high prevalence of dementia (Portugal) and examine its psychometric properties.

**Methods:**

The reliability, structural validity, and criterion validity of the DIS-PT were assessed by applying the scale using a remote measurement web platform. A sample of 105 dementia caregivers completed the DIS-PT and several psychosocial measures, including caregiver burden, anxiety, depression, quality of life, PwD functional independence, and neuropsychiatric symptoms.

**Results:**

The DIS-PT demonstrated good structural validity, with one factor explaining 75% of the total variance. The internal consistency of the scale was high (*α* = 0.802). Most caregivers (65.7%) endorsed at least one item on the DIS-PT (Mdn 2). The caregiver’s desire to institutionalize was significantly associated with the caregiver, care recipient, and contextual variables previously known to affect institutional placement. These included the caregivers’ occupational status, perceived burden, anxiety (but not depression), physical and psychological quality of life, care recipient education, severity of neuropsychiatric symptoms, and cohabitation with the caregiver.

**Discussion:**

This study offers preliminary support for the psychometric quality of the DIS-PT. The scale has practical applications in the early identification of caregivers considering nursing home placement, providing room for intervention in modifiable risk factors that may otherwise lead to the institutionalization of PwD. Remote measurement tools may hold value in assessing caregiving dyads non-intrusively and inexpensively.

## 1 Introduction

Dementia is a significant public health concern due to its high prevalence and substantial economic and social impact on families, healthcare systems, and society (1). Worldwide, the number of persons with dementia (PwD) is estimated at 55 million (2). Among older adults, dementia is the primary cause of dependence and the most frequent reason for institutionalization, primarily due to its significant burden on informal caregivers (3). Within 1 year of diagnosis, approximately 20% of PwD are placed in institutional care, with admission rates increasing to 50% within 5 years and nearly 90% within 8 years (4).

Most PwD prefer to remain in their homes, preserving their familiar environments and social connections (4). Institutionalization has also been linked to poorer health-related quality of life among older persons (5). From a health and social support system perspective, institutional care carries significant financial implications due to its high cost (6). However, for PwD who are severely dependent and exhibit challenging behavioral and psychological symptoms of dementia (BPSD), moving to a long-term care facility may be expedient, as remaining at home can limit the quality of care and impose high levels of stress on informal caregivers. For PwD staying in the community, the costs associated with the disease primarily fall on family and informal caregivers, particularly in low-and middle-income countries (7) and regions where a Mediterranean or familialistic model of care is predominant (8).

Over the past three decades, research has demonstrated that the circumstances under which PwD transition to long-term care are multifactorial. These factors encompass sociodemographic, health-related, and psychological aspects pertaining to PwD and the informal caregiver, as well as contextual factors. Systematic reviews indicate that sociodemographic predictors of nursing home placement pertaining to PwD include being older, unmarried (4, 9, 10), and living alone (4, 9). Regarding the sociodemographic variables of caregivers, conflicting findings have been reported regarding the association between being a spouse or a child of a PwD and the likelihood of nursing home placement (4, 9, 10). Caregivers with higher levels of education, better employment, and higher income seem more likely to place a relative with dementia in long-term care (4, 9).

Sociodemographic factors interact with disease-related and context-of-care factors. Research synthesis studies have shown that greater severity of dementia, higher degrees of cognitive and functional impairment, the presence and severity of BPSD, and a diagnosis of Alzheimer’s disease are associated with an increased risk of institutional placement (4, 9–11). Also, higher caregiver burden and poorer physical and mental health have been associated with an increased likelihood of care recipient institutionalization (4, 9–11), although findings regarding caregiver depression and physical health have been reported to be inconsistent in one review (11). Lower caregiver life satisfaction and lower health-related quality of life are associated with a higher risk of institutional placement (4, 9). Regarding the context of care, studies have inconsistently reported an association between institutional placement and either more or less caregiving hours (4, 10). Community support services have been associated with institutionalization, with studies showing inconsistent findings in both positive and negative directions (4, 9, 11).

In addition to the factors mentioned above, the contemplation of future institutional care by the caregiver, on whom the decision often relies, was suggested as the most important predictor of actual institutionalization for individuals with dementia (4). Caregivers tend to contemplate the institutional placement of PwD long before it happens, suggesting that institutionalization is a gradual process rather than an abrupt event (12). This process is known as the desire to institutionalize. The desire to institutionalize is significantly less researched than actual institutionalization, but it is suggested that it shares similar predictors (4). This thesis is supported by recent research on predicting the desire to institutionalize, which has found that the most consistent predictors of actual institutionalization, such as poor autonomy and frequency of BPSD in PwD, as well as caregivers’ burden, also serve as predictors of the desire to institutionalize (13–15).

The “Desire to Institutionalize Scale” (DIS) (16) is a widely used instrument for assessing the desire to institutionalize among caregivers of PwD, which has consistently demonstrated a strong predictive ability for future institutionalization (12, 17, 18). Although the scale has been translated and adapted into multiple languages in different countries [e.g., Belgium (15), India (19)], it currently lacks adaptation to European Portuguese or application in Portugal. Instruments like the DIS can be valuable for early identification of caregivers considering nursing home placement. They offer an opportunity to address modifiable risk factors that could lead to institutionalization, such as the burden of care. These instruments may also support effective care planning and help prevent the escalation or chronicity of stress that caregivers may experience when transitioning out of their caregiving roles. The DIS is an obvious choice in assessing the desire to institutionalize, given its multiple international adaptations, evidence of psychometric qualities, and extensive use in caregiving and dementia research.

This cross-sectional study aims to translate and adapt the “Desire to Institutionalize Scale” (DIS) (16) into European Portuguese (DIS-PT) and examine its psychometric properties. The reliability, structural, and criterion validity of the DIS-PT are assessed by administering the scale to a sample of Portuguese informal dementia caregivers using a remote measurement platform (iSupport-Portugal). iSupport-Portugal is an online training program for caregivers of PwD initially developed by the World Health Organization and culturally adapted to Portugal (20, 21). The platform is being explored for its potential to remotely assess the health and well-being of caregiver-PwD dyads.

Portugal offers an interesting context to evaluate the desire to institutionalize due to its high prevalence of dementia (21 cases per 1,000 inhabitants) (22) and a significant proportion of overall informal caregivers who report supporting a person with dementia (33%) (23). The familialistic approach to caregiving prevalent in the Mediterranean, where caregiving is influenced by social pressure (8), further adds to the significance of studying the desire to institutionalize in this country.

## 2 Materials and methods

**Figure 1:**
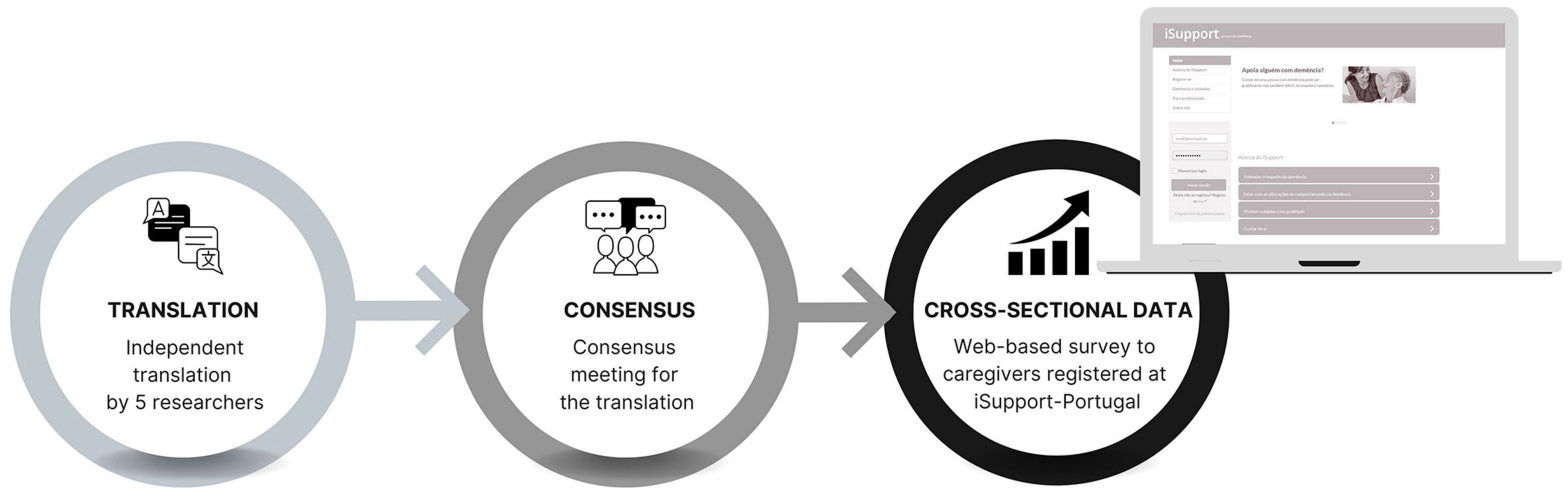
Methodological approach.

A three-step methodological approach was employed, consisting of the following stages: 1. the translation of the DIS into European Portuguese by independent researchers; 2. a consensus meeting for finalizing the translation; and 3. the administration of the DIS-PT to a sample of Portuguese informal caregivers of PwD (see Figure 1).

### 2.1 Translation

The DIS scale comprises 109 words distributed in a two-word initial statement and six items/questions. It was independently translated from American English into European Portuguese by five independent researchers, including i. a professional translator integrated into an R&D center, specialized in terminology and highly experienced with health-related content; and ii. four health specialists with a track record of scientific work on the topics of aging, dementia, and informal caregiving, with backgrounds in psychology (*n* = 2), gerontology (*n* = 1), and health services research (*n* = 1). The five independent translations were analyzed for the level of agreement (see Data analysis).

A consensus meeting was held among all the researchers to discuss their translation choices and reach the final version of the DIS-PT. The translation’s accuracy, clarity, and cultural appropriateness can be validated through several methods. One of the most frequently used is back translation, for which at least two independent bilingual translators are needed, one to translate from the source language into the target language and the other to render the translated text from the target language back into the source language without having access to the actual original text; finally, the two source language texts are compared to verify the level of agreement (24, 25). However, as accepted and valuable as this method may be for correcting problems and validating the first translation, it may also produce equivalence and cultural transfer errors, which subsequent validation processes may not detect. Therefore, this study validated the translation using the committee approach, an already established, valid, and more efficient alternative to back translation (26, 27) in which the original text is independently translated by two or more bilingual individuals who then discuss any differences in translation choices until an agreement is reached, as described in guiding works by linguists (24, 25).

### 2.2 Participants and recruitment

A non-probabilistic sample was drawn from users registered in the eHealth platform isupport-portugal.pt. (see Introduction) from February to June 2023, and participants in the project’s focus groups. The eligibility for participating in the study was limited to individuals who self-declared as i. adults (aged 18 and over), ii. providing unpaid care to iii. a person diagnosed with dementia, iv. living in the community (i.e., not living in a nursing home or other residential institution). The dissemination of iSupport-Portugal was conducted through various channels, including i. the websites and social media platforms of the University hosting the eHealth program or community partners (e.g., National Alzheimer’s Association), ii. media press articles, iii. Scientific or community-targeted seminars, and iv. email contacts of healthcare and social support organizations working with PwD and/or their caregivers. Upon registration at isupport-portugal.pt. to access the eHealth program, caregivers were given complete information and were invited to participate in the research. Non-consent to participate in the study did not hinder the use of the program in any way. A pseudonymization process was implemented, and this study was approved by the Ethics Committee for Health of the Faculty of Medicine of the University of Porto (ref: 76/CEFMUP/2022).

### 2.3 Variables and measures

The study utilized cross-sectional primary data gathered through a web-based survey self-completed by participants. The survey was implemented as a fill-in form on the iSupport-Portugal.pt. intervention-research platform, with the collected data securely stored on the University of Porto. Participants completed the survey after registering on iSupport-Portugal and providing their consent to participate in the research. Caregivers were requested to provide sociodemographic information about themselves and the person receiving care and relevant details regarding the care context. Regarding service utilization, the survey includes questions about services aimed at both PwD (home care service, home health services, day or night centers, respite services, cognitive or occupational therapy) and caregivers (psychoeducational, support or mutual aid groups, mental health consultations, memory cafés). Respondents could suggest additional services that are not listed in the survey.

In addition, the participants completed several psychosocial measures and the European-Portuguese version of the Desire to Institutionalize Scale (DIS-PT). The DIS (16) is a self-report measure comprising six items that assess different stages of contemplating nursing home placement. These stages range from considering the placement of the care recipient to taking active steps toward placement. Caregivers indicate their response using a dichotomous option of “yes” (score = 1) or “no” (score = 0). The scale allows for calculating an overall desire to institutionalize score, which can range from 0 to 6 points. The scale has shown good reliability and predictive ability for actual institutionalization in previous studies (12, 17, 18). Permission to translate and adapt the scale into European Portuguese was obtained from the original author.

The Portuguese versions of widely used measures were administered to study participants to assess their situation. Caregiver burden was evaluated using the Zarit Burden Interview (ZBI-22) (28, 29). This instrument consists of 22 items that are rated on a 5-point scale. The total ZBI score ranges from 0 to 88 points, with higher scores indicating a greater burden. Face, content, ecological, discriminant and convergent validity of the ZBI in its European-Portuguese version have been documented, as well as test–retest reliability (ICC = 0.93, CI 95% 0.88–0.96, *p* < 0.001) and internal consistency (*α* = 0.88) (30). Symptoms of depression and anxiety were measured using the Hospital Anxiety and Depression Scale (HADS) (31, 32). The HADS comprises two subscales, each with 7 items rated on a 4-point scale. Total scores for each subscale range from 0 to 21, with higher scores indicating more severe anxiety or depression symptoms. The European-Portuguese version of the HADS has shown good psychometric properties, similar to the general studies in other languages, including good internal consistency (*α* = 0.76 for anxiety and *α* = 0.81 for depression subscales) (31). Quality of life was assessed using the WHOQOL-BREF (33, 34). The WHOQOL-BREF is a 26-item instrument encompassing four quality-of-life domains: physical, psychological, social relationships, and environment. It also includes items related to the overall quality of life. Each item is scored on a 1-to-5-point scale, and higher values indicate a higher quality of life when computing total scores. The European-Portuguese version of this scale has shown good internal consistency (*α* = 0.92), correlation with the WHOQOL-100 (from *r* = 0.77 to *r* = 0.86 across the QoL domains), good temporal stability (test–retest from *r* = 0.65 to *r* = 0.85 across the QoL domains) and good discriminant validity between patients and controls (33).

The caregivers completed two additional measures regarding the PwD situation. Functional independence in personal activities of daily living (ADL) was assessed using the Barthel Index (35, 36). The version used ranges from 0 (totally dependent) to 20 (totally independent), with individual items scored from 0 to a maximum of 3 points. The European-Portuguese version of this instrument has shown good psychometric properties, including high internal consistency (*α* = 0.96) and strong correlation with the Lawton and Brody scale of instrumental activities (*r* = 0.84) (35). Cut-off points were proposed to classify the person being evaluated into four levels of dependence: total (0–8 points), severe (9–12 points), moderate (13–19 points), and independent (20 points). Neuropsychiatric symptoms were assessed using the Neuropsychiatric Inventory Questionnaire (NPI-Q) (37, 38). The NPI-Q evaluates 12 symptom domains, assessing their presence (yes/no) and severity (mild, moderate, or severe) over the past month. The total NPI-Q severity score ranges from 0 to 36. Additionally, the NPI-Q assesses caregiver distress for each symptom present. The distress scale is rated on a 6-point scale, ranging from “not emotionally stressful” to “extremely stressful,” with the total NPI-Q distress score ranging from 0 to 60. Further information on the psychometric properties of each instrument for its European-Portuguese version can be found in the validation studies cited.

### 2.4 Data analysis

Before the consensus meeting was held, the leading researcher (ST) examined the five independent translations based on the segmentation of the original instrument into meaning units (*n* = 27 units; 1 = initial statement “as caretaker,” and 4–5 units per question, e.g., “have you ever,” “care recipient”). Thereafter, to reach a quantitative indicator of consensus, a working document was prepared in which the translation choices per meaning unit per translator were labeled as (1) identical or (2) most frequent—when all or most translations contained the same words; and (3) different—when the translation choices varied partially or entirely or were omitted by grammatical or stylistic choice or due to idiomatic constraints. In the consensus meeting, it was established that the 135 meaning units in the source text (i.e., 27 meaning units × 5 translators) should be translated into 145 meaning units because two of the questions (Q3 and Q6) had four meaning units each but translated into five in Portuguese due to semantic and syntactic choices. Based on these numbers, the inter-translator agreement was calculated as the number of identical meaning units among translators divided by the total number of meaning units and then multiplied by 100 for a percentage agreement.

The structural validity of the DIS-PT was examined, and to determine the required sample size, the COSMIN guidelines were followed (39). These guidelines recommend a ratio of seven participants per variable, establishing a minimum sample size of 42 (7 × 6 DIS items). A principal component analysis (PCA) based on tetrachoric correlations for binary variables was conducted using the FACTOR software (40). The adequacy of the data to perform the analysis was assessed using the inter-item correlation matrix, the Bartlett test of sphericity (testing the null hypothesis that there is no relationship between the items) (41), and the KMO test (where a value of at least 0.6 is desired) (42). Kaiser’s criterion and Parallel Analysis were used to determine the number of factors to retain since the first retains factors with eigenvalues >1, and the latter compares the size of eigenvalues with those from a randomly generated data set (43).

Item-total correlations and the Kuder–Richardson 20 (KR20) coefficient were utilized to evaluate the internal consistency of DIS-PT. The KR20 coefficient, calculated using the formula $rKR20=\kappa /\kappa -11-\Sigma pq/\sigma \frac{2}{y}\;$ , serves as the dichotomous equivalent to the coefficient alpha. A value of ≥0.70 indicates good internal consistency (44). Item-total correlations were examined to assess the consistency of the items within the scale.

Descriptive statistics were computed for caregiver, PwD, and care context variables and for the desire to institutionalize the care recipient with dementia (DIS-PT). Absolute and relative frequencies, central tendency, and dispersion measures were utilized as appropriate. To assess the criterion validity of the DIS-PT, the associations between the scale’s total scores and caregiver, PwD, and care context variables, which are expected to be correlated with the desire to institutionalize based on previous research (see Introduction), were examined. A criterion can be either a measure of the same construct or any variable demonstrating evidence of correlation with the measure being analyzed (45). Since there is no established golden standard or instrument in European Portuguese for measuring the desire to institutionalize, criterion validity is examined based on the latter. Favorable results for the criterion validity of the DIS-PT would be evidence of an association of the scale’s total scores with the variables that consistently show a significant relationship with institutionalization of a care recipient in the literature (see Introduction and (4)), which is the case of caregiver burden. The study included a feasible sample size of all eligible participants who completed the required measures to explore such associations. A sensitivity analysis was performed using G*Power software to estimate the minimum effect size that could be detected with 80% power (α two-sided = 0.05 and *β* = 0.20) for a sample of 105 participants. For correlations, the analysis was performed using the software for estimating the effect size of a Pearson’s correlation because Spearman’s rank coefficient is computationally identical to the Pearson product–moment coefficient. The minimum detectable effect is 0.26 (medium effect size). For group comparison (Mann–Whitney U test) (α two-sided = 0.05 and *β* = 0.20), the minimum detectable effect would be 0.68 for the less numerically balanced groups compared (male = 23 vs. female = 82) and 0.57 for the more balanced groups (cohabitation, yes = 57 vs. no = 48), so the study would be sensitive to medium and large Cohen’s d effect sizes. The study would not be able to reliably detect correlations smaller than *r* = 0.26 or effects smaller than Cohen’s *d* = 0.57.

Considering the negatively skewed distribution of total scores on the DIS-PT (see Results), Spearman’s Rho Test was used to analyze its associations with continuous or ordinal variables, and the Mann–Whitney U Test was employed to compare two groups. Considering the sample size, categorical variables with more than two levels were dichotomized into theoretically meaningful groups. All *p* values are two-sided with a significance level of 0.05. Statistical analyses were conducted using SPSS version 27 (46) (RRID:SCR_002865).

## 3 Results

### 3.1 Inter-translator agreement

The inter-translator agreement, i.e., the percentage of identical meaning units among translators concerning the total number of meaning units in the target texts, was 67.6% (i.e., 98/145). Some examples of complete agreement (i.e., all the researchers made the same linguistic choice) are the translations of “have you ever” as “*alguma vez*,” “felt” as “*sentiu*,” and “would be better off” as “*estaria melhor*.”

The linguistic choices that differed refer to (1) the person receiving care, in the original text referred to as “patient” and in another version also consulted by the translators (17) as “care recipient,” (2) the care institutions “nursing home,” “boarding home” or the alternative provided by Gallagher et al. (12) “long-term care institution,” (3) verb choices to express (3a) thinking process, i.e., “think” vs. “consider,” (3b) reasoning or argument process, i.e., “discuss” vs. “talk over,” (3c) action taking to change the living arrangement of the PwD, i.e., “move” vs. “place” vs. “institutionalize.”

Overall, semantically broader word choices and a more neutral register were agreed to be better for targeting most of the Portuguese population. Therefore, the meaning units’ final translation options obey this general rule rather than the frequency of translation among the researchers. However, in many cases, the most frequent translation among the researchers was also the closer-to-everyday-language option of translation.

The most frequent noun phrase, “*a pessoa de quem cuida*” (the person you care for), was deemed more appropriate to express the concept of “patient/care recipient” in plain Portuguese. By the same token, the translation of caretaker was “*pessoa que cuida*” (person that cares/takes care) despite not being the most frequent translation. Following the same principle, the closer-to-everyday-language verbs “*pensar*” (think), “*falar*” (talk), and “*colocar*” (place) were chosen over the most frequent researchers’ translations and formal alternatives in European Portuguese “*considerar*” (consider), and “*discutir*” (discuss).

Most of the researchers (*n* = 3) agreed on translating the “residence” concept contained in “nursing home” and “boarding home” as “*lar*” or “*residência sénior*” to be coherent with the Portuguese accommodation models of Residential Facilities for the Elderly (ERPI in Portuguese). Also, the prepositional phrase “*de idosos*” (as in “*lar de idosos*,” literally, elderly’s home) was omitted as it was deemed stigmatizing, and the word “*lar*” (home) is contextually self-explanatory and very well known to the Portuguese.

### 3.2 Characterization of study participants

A sample of 105 eligible self-declared informal caregivers of PwD was considered in this study. Table 1 displays the sociodemographic characteristics of the caregiver and care recipient dyads and the variables concerning the care context. Most caregivers were female, middle-aged (aged 24–83 years), highly educated, and employed at the time of the data collection. Most were offspring caregivers, providing intensive and long-term care, and approximately half lived with the care recipient. While most caregivers were receiving some form of caregiving support and were utilizing community resources for PwD, less than a third (31.4%) were using support services for themselves.

**Table 1:** Summary of caregiver, PwD and care context variables of study participants.

Variable	N	Descriptive statistics
Caregiver factors		
Age (years), M (SD)	105	53.9 (11.7)
Gender, female, n (%)	105	82 (78.1)*
Years of schooling, M (SD)	105	15.1 (4.6)
Marital status, partnered^†^, n (%)	105	66 (62.9)
Occupational status, employed, n (%)	105	68 (64.8)
Relationship with the care recipient	105	
Offspring, n (%)		76 (72.4)
Spouses, n (%)		20 (19.0)
Other, n (%)		9 (8.6)
Perceived burden (ZBI-22), M (SD)	98	36.5 (13.1)
Anxiety symptoms (HADS-A), M (SD)	90	10.2 (4.1)
Depression symptoms (HADS-D), M (SD)	91	7.9 (4.0)
Quality of life (WHOQOL-BREF), M (SD) ^¥^	88	
General		6.8 (1.6)
Physical		25.4 (5.4)
Psychological		21.2 (4.0)
Social relationships		9.6 (2.5)
Environment		28.3 (5.6)
PwD factors		
Age (years), M (SD)	105	78.2 (7.8)
Gender, female, n (%)	105	74 (70.5)
Years of schooling, Mdn (IQR)	98	4 (6.0)
Marital status, partnered^†^, n (%)	103	62 (60.2)
Type of dementia	105	
Alzheimer’s disease, n (%)		49 (46.7)
Vascular dementia, n (%)		18 (17.1)
Frontotemporal dementia, n (%)		14 (13.3)
Dementia with Lewy bodies, n (%)		10 (9.5)
Other/unknown, n (%)		14 (13.4)
Time since diagnosis (years), Mdn (IQR)	104	3.6 (5.1)
Dependence level	105	
Mild, n (%)		13 (12.4)
Moderate, n (%)		35 (33.3)
Severe, n (%)		30 (28.6)
Total, n (%)		27 (25.7)
Functional independence (BI), Mdn (IQR)	85	14 (12.0)
Neuropsychiatric symptomatology (NPI-Q)		
Severity, Mdn (IQR)	85	10 (8.8)
Caregiver distress, Mdn (IQR)	80	12 (12.8)
Care context factors		
Caregiving duration (years), Mdn (IQR)	105	3.2 (4.8)
Hours caring (per week), Mdn (IQR)	105	24 (42.5)
Support for caregiving, Yes, n (%)	105	73 (69.5)
Cohabitation, Yes, n (%)	105	57 (54.3)
Service use^#^	105	
Care recipient, Yes, n (%)		59 (56.2)
Caregiver, Yes, n (%)		33 (31.4)

N/n, number of participants; M, mean; Mdn, median; SD, standard deviation; IQR, interquartile range; ZBI, Zarit Burden Interview; HADS-A, Hospital Anxiety and Depression Scale (anxiety subscale); HADS-D, Hospital Anxiety and Depression Scale (depression subscale); BI, Barthel Index; NPI-Q, Neuropsychiatric Inventory Questionnaire. *One caregiver identifies as non-binary. ^†^Includes married or in a de facto union. ^¥^Reports on raw scores for each QoL domain. ^#^Includes the services described in Materials and methods section.

The care recipients were 78.2 years old on average (see Table 1). Cases of young onset dementia were included (age range at data collection: 45–93 years). The majority were women and had a low level of education. Most were diagnosed with Alzheimer’s disease for a median of 3.6 years (range: <1–16 years). One-third of the caregivers perceived the care recipient’s dependence level as moderate, consistent with the median score on the Barthel Index (see Table 1 and Methods for the proposed cut-offs). The sample, however, was highly diverse concerning the degree of dependence, as indicated by the distribution of dependence levels and by total scores on the Barthel Index ranging from the minimum to the maximum possible (range: 0–20 points).

Among the sample, 94.1% of caregivers reported at least one neuropsychiatric symptom by the PwD, with an average of 5 symptoms (SD 3.0, range: 0–12). The most reported neuropsychiatric symptoms were apathy (82.4%), depression (50.6%), and appetite changes (55.3%), while euphoria was the least reported (12.9%). The average severity score for positive symptoms was higher for apathy (*n* = 70, M 2.21, SD 0.66), motor disturbance (*n* = 35, M 2.09, SD 0.74), and delusions (*n* = 34, M 2.09, SD 0.71), and lower for euphoria (*n* = 11, M 1.5, SD 0.7).

The median caregiver distress for positive symptoms was relatively low on the scale, with a possible maximum of 60 (median 12, IQR 12.8, range: 1–40), which is consistent with previous research [e.g., (47)]. On average, symptoms causing more distress to caregivers were agitation/aggression (*n* = 31, M 2.84, SD 0.86), delusions (*n* = 34, M 2.79, SD 0.91), and anxiety (*n* = 34, M 2.79, SD 0.88). The NPI-Q caregiver distress scale positively correlated with the ZBI-22 total scores (*r*_s_ = 0.323, *p* = 0.003).

Caregivers presented significant levels of burden (see Table 1), as scores of ≥21 have typically been considered indicative of the presence of burden (29). For anxiety symptoms, 27.8% of caregivers would be classified as borderline cases and 44.4% as abnormal cases, i.e., with clinical anxiety symptomatology. Regarding depression, 30.8% showed borderline values, and 22.0% showed abnormal values. Transformed scores (0–100 scale) for the WHOQOL-BREF reveal that the social relationships domain is the lowest assessed (M 54.7, SD 20.8) compared to the physical (M 65.8, SD 19.2), psychological (M 63.3, SD 16.5), and environmental health domains (M 63.4, SD 17.5), as well as general health (M 60.5, SD 19.5).

### 3.3 Structural validity of DIS-PT

The correlation matrix for the 6 items of the DIS revealed several coefficients above 0.3 (inter-item *r*_s_ = 0.19–0.61), suggesting that performing a PCA would be appropriate in the dataset (48). Both Bartlett’s test of sphericity (χ^2^ = 1164.7, *df* = 15, *p* < 0.001) and the KMO test value (0.73; exceeding the recommended minimum of 0.6) (42) supported the factorability of the correlation matrix. The analysis resulted in 1 factor with an eigenvalue >1.00 (4.51), explaining 75% of the total variance. Parallel analysis based on PCA revealed only one component with an eigenvalue exceeding the corresponding criterion value for the randomly generated data matrix of the same size (500 replications). Hence, the analysis supports a unifactorial structure of DIS-PT. Table 2 presents the factor loadings for each item.

**Table 2:** Bias-corrected and accelerated (BCa) bootstrap 95% confidence intervals for loading values.

Item	Item description	Loadings	BCa confidence interval
1	Ever considered a nursing home or boarding home?	0.952	0.873–0.989
2	Ever felt patient better off in nursing or boarding home?	0.821	0.593–0.931
3	Ever discussed institutionalization with family or others?	0.925	0.800–0.977
4	Ever discussed institutionalization with patient?	0.775	0.529–0.894
5	Be likely to move patient?	0.845	0.686–0.931
6	Steps toward placement?	0.872	0.723–0.947

BCa, Bias-corrected and accelerated.

### 3.4 Reliability analysis: internal consistency

The reliability analysis for the DIS-PT yielded a reliable KR20 value of 0.802. Considering the limited number of items in the scale, this coefficient is good and was not enhanced by removing any item (see Table 3). Corrected item-total correlations ranged from 0.426 (item 4) to 0.712 (item 1).

**Table 3:** Item-total (corrected) correlations and Cronbach’s α if an item is deleted for the DIS-PT reliability analysis.

Item	Corrected item-total correlation	Cronbach’s α if deleted
1	0.712	0.733
2	0.457	0.793
3	0.635	0.753
4	0.426	0.799
5	0.555	0.773
6	0.567	0.770

### 3.5 Caregiver’s desire to institutionalize the person with dementia

Most caregivers (65.7%, *n* = 69) endorsed one or more items on the DIS-PT, while 34.3% (*n* = 36) scored 0, indicating no desire to institutionalize the relative with dementia. The median score among caregivers who responded positively to at least one item of the DIS-PT was 3 (IQR 3, range 1–6), reflecting mild intent. An overall median score of 2 (*n* = 105, IQR 4) was obtained. An item-by-item analysis (see Table 4) shows that more than half of the caregivers (54.3%) had already discussed institutionalization with family or others. Although 41% considered institutionalization likely, most never felt the person cared for would be better off in a nursing home (*n* = 46, 60.5%). Most caregivers had not talked to the PwD about transitioning to a long-term care facility. Among those who had taken steps toward placement, less than half (48.3%) had had such conversations, and more than half never believed their relative would be better off in a nursing home (*n* = 17, 58.6%). Having had or not a conversation with the person in care about transitioning to a care facility was not associated with their degree of functional independence (*U* = 664.5, *z* = −0.077; *p* = 0.939), dependence level as perceived by the caregiver [χ^2^_(1, *N* = 105)_ = 0.069, *p* = 0.793], or severity of neuropsychiatric symptoms (*U* = 540.5, *z* = −1.252; *p* = 0.211).

**Table 4:** Caregiver’s desire to institutionalize a PwD assessed with DIS-PT.

DIS items		Yes, n (%)
	As a caregiver	
1	Ever considered a nursing home or boarding home?	48 (45.7)
2	Ever felt patient better off in nursing or boarding home?	23 (21.9)
3	Ever discussed institutionalization with family or others?	57 (54.3)
4	Ever discussed institutionalization with patient?	25 (23.8)
5	Be likely to move patient?	43 (41.0)
6	Steps toward placement?	29 (27.6)

Frequencies per item (*N* = 105).

### 3.6 Criterion validity of DIS-PT: associations with caregiver, PwD, and care context variables

The criterion validity of the DIS-PT was evaluated by investigating the associations between the scale’s total scores and the caregiver, PwD, and care context variables. For the analyses, variables anticipated to be correlated with the willingness to institutionalize, as indicated by previous research (see Introduction), were chosen along with the age, gender, and education of both the PwD and the caregiver (see Table 5).

**Table 5:** Associations of the desire to institutionalize (DIS-PT total score) with caregiver, PwD, and care context variables (*N* = 105).

Variable	Mdn (IQR, mean rank)/Spearman’s r*	*p*
Caregiver factors		
Age (years)^a^	−0.043	0.663
Gender^b^		0.928
Female	2 (4, 52.6)	
Male	2 (4, 52.0)	
Years of schooling^a^	−0.087	0.376
Occupational status^b^		**0.005**
Employed	3 (3.8, 59.0)	
Not employed	0 (3, 42.1)	
Relationship with the care recipient^b^		0.317
Offspring	2 (4, 54.8)	
Other	2 (3, 48.3)	
Perceived burden (ZBI-22)^a^	0.366	**<0.001**
Anxiety symptoms (HADS-A)^a^	0.250	**0.018**
Depression symptoms (HADS-D)^a^	0.031	0.774
QoL (WHOQOL-BREF)—general^a^	−0.151	0.158
QoL (WHOQOL-BREF)—physical^a^	−0.238	**0.025**
QoL (WHOQOL-BREF)—psychological^a^	−0.237	**0.027**
QoL (WHOQOL-BREF)—social relationships^a^	−0.067	0.531
QoL (WHOQOL-BREF)—environment^a^	−0.136	0.205
PwD factors		
Age (years)^a^	−0.133	0.177
Gender^b^		0.937
Female	2 (4, 52.1)	
Male	2 (4, 51.6)	
Years of schooling^a^	−0.208	**0.040**
Marital status^b^		0.440
Partnered	2 (3, 50.2)	
Not partnered	2 (4, 54.7)	
Type of dementia^b^		0.934
Alzheimer’s disease	2 (4, 52.7)	
Other	2 (4, 53.2)	
Functional independence (BI)^a^	−0.080	0.467
Neuropsychiatric symptoms (NPI-Q), severity^a^	0.224	**0.041**
Care context factors		
Hours caring (per week)^a^	−0.177	0.071
Cohabitation^b^		**0.003**
Yes	1 (3, 45.2)	
No	3 (3, 62.3)	
Service use (PwD or caregiver)^b^		0.811
Yes	2 (4, 52.6)	
No	2 (4.5, 54.2)	

Mdn, median; IQR-interquartile range; ZBI, Zarit Burden Interview; HADS-A, Hospital Anxiety and Depression Scale (anxiety subscale); HADS-D, Hospital Anxiety and Depression Scale (depression subscale); BI, Barthel Index; NPI-Q, Neuropsychiatric Inventory Questionnaire; QoL, Quality of life. *Mdn, IQR, and mean rank are presented for categorical variables; Spearman’s r values are presented for continuous/ordinal variables. ^a^Tested by Spearman’s Rho Test. ^b^Tested by Mann–Whitney U Test; values in bold highlight the statistically significant associations (*p* < 0.05).

Regarding the caregiver factors, i. occupational status, ii. perceived burden; iii. anxiety but not depression, and iv. physical and psychological QoL, but no other QoL domains were significantly associated with the desire to institutionalize the relative with dementia. Caregivers who were employed presented higher scores on the DIS-PT when compared to those who were not (*U* = 853, *z* = −2.787; *p* = 0.005). Moderate positive correlations between ZBI-22 total scores (*r*_s_ = 0.366, *p* = <0.001) and HADS-A (*r*_s_ = 0.250, *p* = 0.018) with DIS-PT total scores revealed that caregivers experiencing higher burden and anxiety symptoms are more willing to place the relative with dementia in institutional care. Inversely, caregivers scoring higher on physical (*r*_s_ = −0.238, *p* = 0.025) and psychological QoL domains (*r*_s_ = −0.237, *p* = 0.027) reported less willingness to resort to institutional care.

Regarding the variables pertaining to the PwD, i. the care recipient’s education was negatively correlated with the caregivers’ desire to institutionalize (*r*_s_ = −0.208, *p* = 0.040), while ii. the presence and severity of neuropsychiatric symptoms (NPI severity score) were positively associated with such desire (*r*_s_ = 0.224, *p* = 0.041). The functional independence of the PwD did not yield significant associations with the caregivers’ desire to institutionalize.

The desire to institutionalize a relative with dementia was higher among non-cohabiting caregivers compared to those living with the PwD (*U* = 923, *z* = −2.937; *p* = 0.003).

## 4 Discussion

### 4.1 Main findings and contributions

This study focused on the translation, adaptation, and examination of the psychometric properties of the European-Portuguese version of the Desire to Institutionalize Scale (DIS-PT) (16). The scale demonstrated good structural validity (one factor explaining 75% of the total variance), high internal consistency (*α* = 0.802) and association with caregiver, care recipient, and contextual variables previously known to affect institutional placement, including the caregivers’ occupational status, perceived burden, anxiety, physical and psychological quality of life, care recipient education, severity of neuropsychiatric symptoms, and cohabitation with the caregiver. Overall, the study offers preliminary support for the psychometric quality of DIS-PT.

Institutionalizing a relative in care can be considered a critical life event influenced by multiple factors related to the caregiver, the PwD, and the overall context. This event requires the dyads’ behavioral, cognitive, and emotional adaptation. Caregivers often experience increased stress when transitioning out of caregiving, wishing they could have delayed the institutionalization of the PwD (49). The contemplation of institutional placement by the caregiver, a key predictor of actual institutionalization (4), is a complex and lengthy stage. This stage offers an opportunity for interventions to help smooth or delay the institutionalization process.

This study offers a valuable contribution by providing a tool—DIS-PT—to identify caregivers contemplating transitioning out of caregiving and, potentially, PwD at a higher risk of institutional placement. As far as the authors know, the adapted version of this instrument is the only tool available in Portugal to assess the desire to institutionalize. The DIS is a short scale that can be readily incorporated into research protocols and routine health and social care assessments for caregivers of PwD. Information provided by the DIS-PT might be used to intervene in modifiable factors that influence the desire to institutionalize, and that otherwise would increase the likelihood of early placing the PwD in institutional care.

Given the scarcity of healthcare and social service resources, scaling up the assessment of the desire to institutionalize among dementia caregivers and referring them to pre-emptive interventions on time is challenging. One innovative aspect of this study is that the desire to institutionalize and related variables were assessed with minimal resource usage using an online intervention research platform (iSupport-Portugal). Such remote measurement tools have been increasingly explored as alternatives to conventional assessment measures, enabling real-time and longitudinal monitoring of health-related behavior inexpensively and unobtrusively (50). The remote measurement platform used in this study is proving its usefulness for collecting data on caregiver-care recipient dyads. As political investments across Europe in closing the digital divide begin to yield effects and digital natives assume the role of caregivers, the platform’s capabilities can be further enhanced.

Three key findings emerged from this study concerning the psychometric properties of the DIS-PT. First, the European-Portuguese version of this scale demonstrated good structural validity, with one factor being extracted and explaining 75% of the total variance. Structural validity pertains to the extent to which the scores of an instrument accurately reflect the underlying dimensionality of the measured construct. The DIS-PT exhibits good structural validity with more than 50% of the variance being explained (51). All scale items demonstrated factor loadings above 0.70, considered excellent (52). Second, the internal consistency of the DIS-PT was found to be high (*α* = 0.802). When compared to previous research on the DIS, the reliability coefficients for the DIS-PT demonstrated comparable results [e.g., KR20 alpha of 0.71 for the original study of the DIS (16), 0.82 in Pruchno et al. (53), or 0.64–0.76 among USA ethnic groups in McCaskill et al. (17)]. Third, the DIS-PT demonstrated good criterion validity by correlating with variables consistently reported in the literature associated with the desire to institutionalize. Bivariate analyses revealed that the desire to institutionalize was positively associated with several caregiver-related variables. As found in previous studies, these include a higher burden and being employed (12, 14, 15, 18).

Additionally, the caregiver’s desire to institutionalize was positively associated with higher anxiety and lower perceived physical and psychological quality of life. Previous literature on the predictors of actual institutionalization has concluded similarly (4, 9). Symptoms of depression were found not to be associated with the desire to institutionalize, which is also consistent with a recent review on predictors of institutional placement (11).

Positively associated factors related to PwD included the severity of neuropsychiatric symptoms also observed by Kapoor et al. (14) and Vandepitte et al. (15) but not the level of functional independence [contrasting with conclusions by Colucci et al. (13) and Kapoor et al. (14)]. This finding, however, was not at odds with previous literature (18, 19), including research showing that dementia stages and activities of daily living have indirect effects on caregiver burden through behavioral and psychological symptoms of dementia, which exert a mediating effect (54). This study also revealed a negative association between the desire to institutionalize and the education level of the PwD. Unlike caregivers’, the care recipients’ education level has not been emphasized in research on the desire to institutionalize. This association is likely to be related to family income, which has been previously shown to be correlated with the institutionalization of PwD (4, 9).

This study also suggests that among the context of care factors, cohabitation with the person in care serves as a protective factor against the desire to institutionalize. In line with Vanderpitte’s findings (15), this suggests that cohabitation has a greater influence on the desire to institutionalize than the relationship within the dyads (i.e., spouse/children) or the marital status of PwD, which did not show independent associations. These variables are associated at a certain level but are distinctive.

Some other variables significantly associated with the desire to institutionalize in earlier studies, such as caregivers’ age (14, 15, 18) or education (15), did not show similar associations in the current one. This result might be attributed to a self-selection bias, where the participants in this study tend to be highly educated and internet-savvy. Moreover, the literature on the association between community services use and the desire to institutionalize has yielded inconsistent findings and yields non-significant associations in this study.

This study comprehensively assessed a wide range of variables potentially influencing the desire to institutionalize and actual institutionalization using well-described and validated tools. Such assessments are valuable in describing the profile of the caregiver-care recipient dyads and should be further explored in future research. The primary objective of this study was not to derive an explanatory model of the desire to institutionalize. The associations were explored primarily to assess the criterion validity of the DIS-PT. Nonetheless, they offered insights into the association of the desire to institutionalize with caregivers, PwD, and care context variables, reinforcing the role of potentially modifiable factors on the desire to institutionalize, such as burden and anxiety, which may be addressed through caregiver interventions.

This study made a significant contribution by examining the desire to institutionalize a relative with dementia in a sample of caregivers in a country with a high prevalence of dementia and reliance on informal care (see Introduction). To the best of the authors’ knowledge, there is no recent account of the desire to institutionalize in a sample of Portuguese dementia caregivers, although perseverance time, a distinctive concept, has been described (55). The proportion of caregivers endorsing at least one item on the DIS (65.7%) is in alignment, albeit slightly higher, with those found in other studies, ranging from 50 to 63.4% (12, 15, 18, 19). An item-by-item examination of the DIS-PT answers revealed that while most caregivers (54.3%) had already discussed institutionalization with family or others, this topic had rarely been discussed with the PwD themselves, and this was not associated with the reported dependence level of the person in care. Significantly, among caregivers considering institutionalization to be likely, and even those already taking steps toward placement, most did not believe that the PwD would be better off in institutional care (see Results). This duality of envisioning or taking steps toward an action that was not considered to be the most suitable for a loved one may increase the sense of guilt and put caregivers under additional risk of stress after transitioning out of caregiving. This fact further highlights the importance of identifying caregivers contemplating institutional placement to offer interventions that might minimize deleterious outcomes post-institutionalization.

### 4.2 Limitations and future research

Findings from this research should be interpreted in the light of its limitations. Recruitment of caregivers was not random and was carried out by disseminating the e-intervention program iSupport-Portugal, increasing the chances of volunteer bias. Caregivers seeking education and support may be more inclined to keep their relatives with dementia at home. Moreover, the sample consisted of digitally literate caregivers. As education level is a well-known determinant of internet usage (56), highly educated caregivers may be overrepresented (63.8% of caregivers in this study have over 12 years of schooling). Portuguese dementia caregivers have predominantly been described as low educated [e.g., (55)]; however, unambiguous national statistics are currently unavailable.

On the other hand, participants in caregiving studies are often recruited through support projects that are less accessible to employed, younger, and more educated caregivers. This study may have reached caregivers typically overlooked and others relying on conventional recruitment methods. Finally, the criterion validity analysis in this study was constrained by the absence of a validated scale to compare with the DIS-PT. As a result, based on previous research, this analysis was limited to examining associations with variables expected to be correlated.

In future research, the same platform used to collect this cross-sectional data will be used to follow up a cohort of caregivers over time to track actual institutionalization. This action will enable the researchers to examine how the sociodemographic and psychosocial variables collected at baseline predict the outcome. The follow-up will also allow for an assessment of the predictive ability of the DIS-PT concerning the actual institutionalization of PwD.

### 4.3 Final remarks

Various strategic documents have emphasized the policy principles of aging at home and supporting informal caregivers (57, 58). These same principles are reflected in national documents, such as the Portuguese Health Strategy for Dementia and the Informal Caregiver Statute (Law nr 100/2019). Whether there are sufficient resources to meet the needs of PwD and ensure they can remain at home with good quality care and quality of life depends on various factors, including the availability of informal caregivers. Understanding the desire to institutionalize among caregivers is of utmost importance for effective care planning for the caregiver-care recipient dyads. This knowledge should inform the design of interventions aimed at smoothing care transitions. It may also inform interventions targeting modifiable factors influencing the desire to institutionalize, thereby increasing the chances for care recipients to age in place.

## Data availability statement

The raw data supporting the conclusions of this article will be made available by the authors, without undue reservation.

## Ethics statement

The studies involving humans were approved by the Ethics Committee for Health of the Faculty of Medicine of the University of Porto (Rapport: 76/CEFMUP/2022). The studies were conducted in accordance with the local legislation and institutional requirements. The participants provided their written informed consent to participate in this study.

## Author contributions

ST: Conceptualization, Formal analysis, Funding acquisition, Investigation, Methodology, Project administration, Writing – original draft, Writing – review & editing, Data curation. MN: Formal analysis, Writing – original draft, Writing – review & editing, Methodology. OR: Methodology, Writing – review & editing. SA: Funding acquisition, Methodology, Writing – review & editing. AFr: Methodology, Writing – review & editing. AFe: Methodology, Writing – review & editing. CP: Conceptualization, Funding acquisition, Methodology, Project administration, Writing – review & editing.
